# Effects of hippotherapy on motor function of children with cerebral palsy: a systematic review study

**DOI:** 10.1186/s13052-024-01715-9

**Published:** 2024-09-19

**Authors:** Panagiotis Plotas, Angelos Papadopoulos, Evangelia-Maria Apostolelli, Eleni Vlachou, Foteini Gazou, Ioanna Zogopoulou, Ioanna Katsaidoni, Ioanna Panagiotopoulou, Sofia Paraskevi Paparouna, Nikolina Silavou, Katerina Fragkiadaki, Eirini Tsiamaki, Sotirios Fouzas, Xenophon Sinopidis, Nikolaos Trimmis

**Affiliations:** 1 Laboratory of Primary Health Care, School of Health Rehabilitation Sciences, University of Patras, Patras, 26504 Greece; 2 Department of Speech and Language Therapy, School of Health Rehabilitation Sciences, University of Patras, Patras, 26504 Greece; 3 General Children’s Hospital of Patras “Karamandaneio”, Patras, 26331 Greece; 4 Department of Neurology, School of Medicine, University of Patras, Patras, 26504 Greece; 5 Department of Pediatrics, School of Medicine, University of Patras, Patras, 26504 Greece; 6 Department of Pediatric Surgery, School of Medicine, University of Patras, Patras, 26504 Greece

**Keywords:** Hippotherapy, Systematic review, Alternative physical therapy, Horse-riding simulator, Motor skills, Cerebral palsy, Effectiveness, Children

## Abstract

Cerebral palsy includes a spectrum of neurodevelopmental disorders caused by brain damage, leading to impairment of movement, posture, and balance for a lifetime. There are many therapeutic interventions for cerebral palsy. One of them is hippotherapy, an alternative physical therapy. It is a type of equine-assisted activity and therapy where children with cerebral palsy and motor function skills interact with a horse. We aimed to study the effects of hippotherapy, as an alternative therapy, on the motor function of children with cerebral palsy. We performed a review of the latest literature on hippotherapy and cerebral palsy. The criteria we used were specific keywords, publication date, age of the subjects/studied population, and article type. The outcome of our research resulted in ten relevant studies. The findings demonstrate improvements in various aspects of motor function – more specifically in gross motor function skills, balance, coordination, gait parameters, and muscle strength. Two of ten studies used the Horse-Riding Simulator with significant improvements in postural control in sitting, motor function, and hip abduction range of motion. Hippotherapy can improve motor function skills of children between 2 and 14 years of age with cerebral palsy, affecting their well-being and overall quality of life. It can have a positive effect either as a standalone treatment or as a part of other traditional therapies. Extended research is needed to assess whether hippotherapy may present significant long-term changes in motor skills in children with cerebral palsy.

**Supplementary Information:**

The online version contains supplementary material available at 10.1186/s13052-024-01715-9.

## Introduction

Cerebral palsy (CP) involves a group of neurodevelopmental disorders caused by brain damage, leading to impairment of movement, posture, and balance [1–4]. The main motor disorder is frequently accompanied by concomitant impairment of sensation, cognition, communication, perception, behavior, and/or seizure disorder [5, 6] linked to decreased quality of life [7]. Therefore, the ability to walk, eat, swallow, and talk is often impaired [8, 9]. Approximately 50% of children diagnosed with CP experience a speech disorder [6], while approximately 33% are unable to communicate verbally [5, 6]. It affects approximately 2–3 out of every 1,000 births (0.2–0.3%), manifests during early childhood, and continues throughout an individual’s lifetime [1, 4]. Generally, diagnosis occurs between the ages of 12 and 24 months [3].

The effective management of children diagnosed with CP necessitates the implementation of an interdisciplinary approach, which leverages the collective expertise of various specialists across diverse disciplines [10, 11]. A wide range of therapeutic interventions, including non-traditional or complementary and alternative medicine, are commonly employed to address the needs of children with CP [10]. Moreover, studies have demonstrated the effectiveness of conventional physiotherapy [12, 13], speech therapy [8] and occupational therapy interventions [14, 15] in enhancing the functional abilities of children diagnosed with CP [10]. As mentioned above, various therapeutic interventions have been explored for the improvement of the motor function of children with CP [10, 12–15] and one such is hippotherapy, which is a form of alternative therapy aided by interaction with horses [16–19]. Hippotherapy consists of the words meaning “horse”- “hippos”, and “treatment”-“therapy” in the ancient Greek language [16, 20]. It was mentioned at first in Hippocrates’ works. Nevertheless, prior to the 1960s, a standardized protocol for the discipline had not yet been established [16]. In the 1960s, it was used as an adjunct to traditional physical therapy in European countries (Germany, Austria, and Sweden) [16]. Moreover, these countries used the horse in physical therapy the term “hippotherapy,” that it was introduced into the medical literature, and after 1992 the American Hippotherapy Association (AHA) established an official, and international protocol [16].

At this point we need to emphasize the distinction between hippotherapy and therapeutic horseback riding, a common source of confusion. As mentioned above, hippotherapy is a therapeutic approach used in physiotherapy and occupational therapy, which aims to treat people with neurological problems and other forms of disabilities that affect mainly motor function and balance [21, 22]. On the other hand, therapeutic horseback riding is a form of recreational activity that is practiced by trained staff and its objective is to improve body stature and the rider’s sense of balance [21, 22]. Specifically, horseback riding offers a range of benefits to its riders, including effective sensory stimulation achieved through the horse’s rhythmic and repetitive movements [16, 20]. The horse’s movement mimics the typical locomotive patterns of the human pelvis during ambulation [16, 20]. The ability to measure sensory stimulation through variations in horse gait allows therapists to integrate these measurements with clinical therapies, to achieve desired outcomes [16, 20]. Hippotherapy improves balance, muscle control, movement, posture, and recuperation times [16, 20, 23]. Horses’ careful and regular exercises help strengthen paraspinal muscles [16, 20, 23]. The horse’s varied swinging rhythm puts twice as much force on a patient’s pelvic girdle bones as another patient’s stride and this engaging treatment technique improves patients’ compliance and enthusiasm [16, 20, 23].

This systematic review aims to record, study, and delve into the effects of hippotherapy on the motor function of children with CP, based on the findings of a number of relevant studies of the latest Literature.

## Materials and methods

**Fig. 1:**
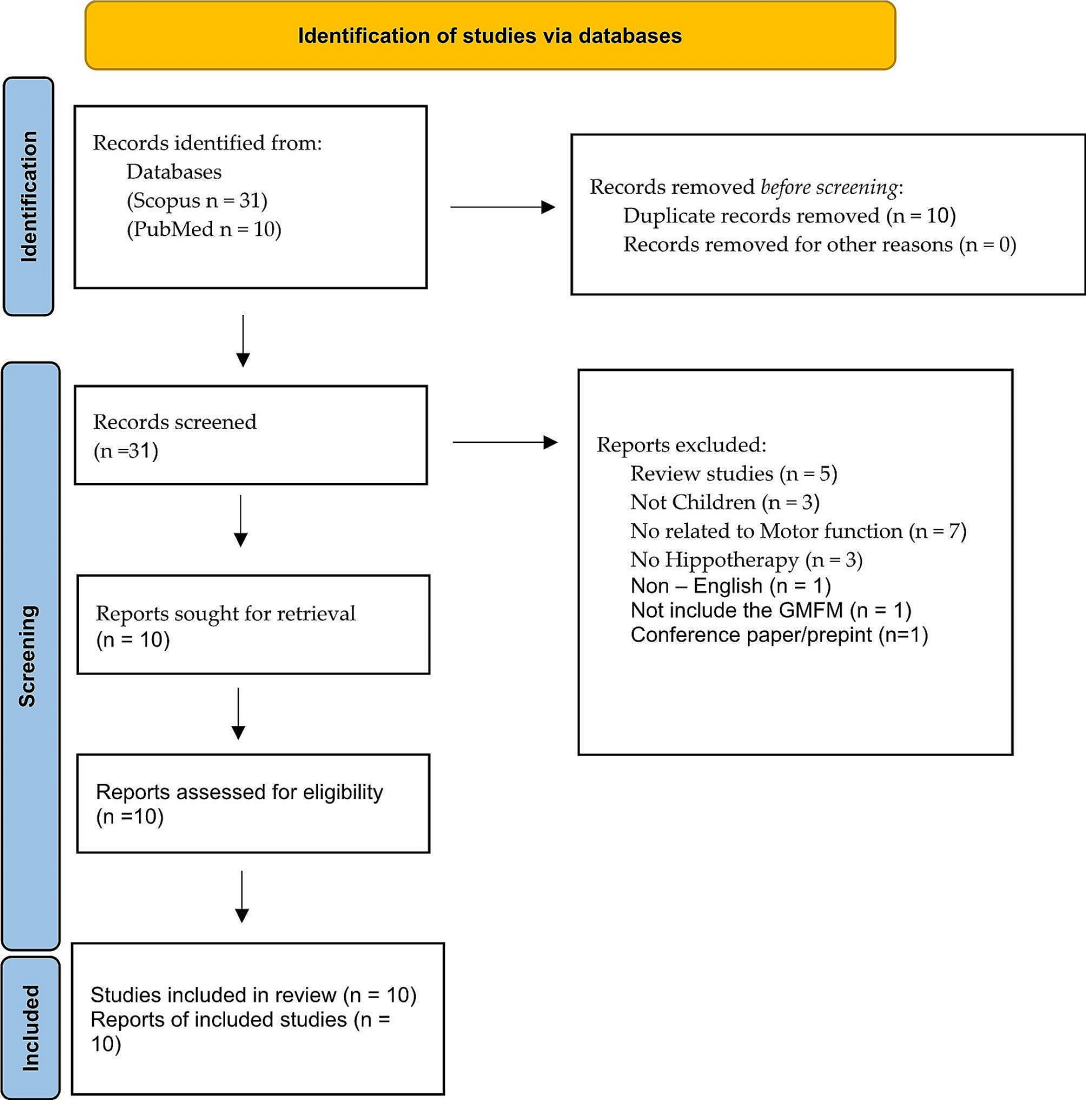
PRISMA 2020 flow diagram for new systematic reviews, which included searches of databases

A broad search was conducted in PubMed and Scopus databases to mine the articles used for this systematic review. More specifically, we used relevant keywords such as “hippotherapy”, “children”, “cerebral palsy” and “motor function”, Scopus 31 articles using this query: (TITLE-ABS-KEY ( hippotherapy ) AND TITLE-ABS-KEY ( children ) AND TITLE-ABS-KEY ( cerebral AND palsy ) AND TITLE-ABS-KEY ( motor AND function ) ) AND PUBYEAR > 2014 AND PUBYEAR < 2023 AND ( LIMIT-TO ( DOCTYPE, “ar” ) ) AND ( LIMIT-TO ( LANGUAGE, “English” ) ). PubMed results included 10 articles, and all were duplicated with Scopus results. The PRISMA Flow Diagram was used to record different stages of the literature search process (Fig. 1) [24].

The criteria used included specific keywords, publication date, age of the subjects/studied population, and article type. Furthermore, the publication period was limited to the last eight [8] years (from 2015 to 2023) to include the latest literature regarding the topic and choose sources for children ages 0 to 14. We excluded review studies and conference papers to avoid overlapping studies and included only original research articles that used real horses or a horse simulator.

The filtered search yielded a total of 41 articles. After initial evaluation was performed, eleven [11] articles were singled out based on their relevance to the research topic (the effects of hippotherapy on the motor function of children with CP). Eventually, the methodologies, findings, and implications of these were critically reviewed. It is important to mention that an additional factor that led to the final selection of articles was the usage of the measurement tool(s) used for the classification of motor function impairments of children with CP, such as the Gross Motor Function Classification System (GMFCS) [25, 26]. GMFCS is a five-level classification system that includes four age groups (< 2y, 2-4y, 4-6y, and 6-12y) [25, 26]. In addition, for the randomized control studies, the Physiotherapy Evidence Database (PEDro) scale [27] used to evaluate the methodological quality of the included studies (Table 1). In literature, this instrument is widely used worldwide with recognized reliability and validity [28, 29]. Two evaluators conducted a separate analysis for each study until a classification was confirmed. Conflicts were resolved after a broad discussion between the evaluators. Mendeley reference manager software was used to remove duplicates.

**Table 1:** The main features of reviewed studies

Studies	Participants/ Therapy Duration	Frequency	Age range (years)	GMFCS level	Assessment tool/method	Study Findings
Matusiak-Wieczorek et al., 2016 [32]	39/ 12 weeks	30 min/session 1–2/week	6–12	I-II	GMFM SAS	The control of position and function of almost every assessed body part (head, trunk, feet, arms, hands) of the participants showed improvement.
Kwon et al., 2015 [33]	92/ 8 weeks	30 min/session 2 /week	4–10	I-IV	GMFM-88, GMFM-66, PBS	Significant improvements in motor function and balance of children who received hippotherapy intervention.
Deutz et al., 2018 [34]	73/ 16–20 weeks (twice)	1–2/week	5.8–12.4	II-IV	GMFM-66, CHQ-28, KIDSCREEN- 27	No significant changes were found in GMFM total scores, yet there was a notable increase in GMFM dimension E: walking, running, and jumping
Lucena-Antón et al., 2018 [35]	44/ 12 weeks	45 min/session 1 /week	3–14	IV-V	MAS	Suggest combining conventional therapy with hippotherapy in a specific frequency to improve significantly hip adductor spasticity in children with CP and positively affect motor skills.
Chinniah et al., 2020 [36]^1^	30/ 12 weeks	15 min/session 3 /week	2–4	I-III	GMFS-88	i. In different stages of the treatment, both groups gradually demonstrated improvement in their sitting motor function according to the GMFM scale. ii. Those who received the combined therapy improved even better during the research period. iii. Riding the HRS appears to improve postural control in sitting and motor function of children with spastic diplegia.
Hemachithra et al., 2020 [37]^1^	24/ -	30 min/session^1^	2–4	GMFCS I-III MAS 1–3	MAS PROM	Children subjected to HRS show reduced muscle tone and improved hip abduction range of motion.
Champagne et al., 2017 [38]	13/ 10 weeks	30 min/session, 1 /week	4–12	I-II	BOT2-SF, GMFS-88	Hippotherapy can benefit gross motor function in children at GMFCS levels I and II.
Seung Mi et al., 2019 [39]	146/ 8 weeks (Total of 16 sessions)	30 min/session, 2/week	3–11	I-IV	GMFS-66, GMFS-88, PBS	The children with CP, GMFCS level I–III had improved postural control in sitting
Ali et al., 2022 [40]	60/ 12 weeks	1 h/session, 3/week	3–5	III - IV	GMFM-88	Hippotherapy is more effective than whole-body vibration in improving sitting function and abdominal muscle thickness.
Prieto et al., 2021 [41]	20/ 16 weeks	30-minute hippotherapy sessions 1–2/week	2 to 5 years and 11 months	II, III, IV, V	GMFM, Pediatric Evaluation of Disability Inventory	Hippotherapy improved the gross motor function and functional performance of children with cerebral palsy, regardless of the weekly frequency of the sessions.

The quality of all the non-randomized studies included in the analysis was evaluated using the methodological index for nonrandomized studies (MINORS) [30]. Each study was assigned a global score based on this assessment. Specifically, the MINORS score is calculated by summing the scores of individual items (ranging from zero to two for each item. The maximum score is 24 for comparative studies and 16 for noncomparative studies [30, 31]. More details are shown in Table 2.

**Table 2:** PEDro Scale outcomes

	Studies						
**Criteria**	(37)	(35)	(33)	(40)	(36)	(34)	(41)
1. eligibility criteria were specified	YES	YES	YES	YES	YES	YES	YES
2. subjects were randomly allocated to groups (in a crossover study, subjects were randomly allocated an order in which treatments were received)	YES	YES	YES	YES	YES	YES	YES
3. allocation was concealed	YES	NO	NO	NO	YES	NO	YES
4. the groups were similar at baseline regarding the most important prognostic indicators	YES	YES	YES	YES	YES	YES	YES
5. there was blinding of all subjects	NO	NO	YES	NO	NO	NO	YES
6. there was blinding of all therapists who administered the therapy	NO	NO	NO	NO	NO	NO	NO
7. there was blinding of all assessors who measured at least one key outcome	YES	YES	YES	NO	NO	YES	NO
8. measures of at least one key outcome were obtained from more than 85% of the subjects initially allocated to groups	YES	YES	YES	YES	YES	NO	YES
9. all subjects for whom outcome measures were available received the treatment or control condition as allocated or, where this was not the case, data for at least one key outcome was analyzed by “intention to treat”	YES	YES	NO	YES	NO	NO	YES
10. the results of between-group statistical comparisons are reported for at least one key outcome	YES	YES	YES	YES	YES	YES	NO
11. the study provides both point measures and measures of variability for at least one key outcome	YES	YES	YES	YES	YES	NO	YES
**Total Score**:	8	7	7	6	6	4	7

**Table 3:** Methodological index for non-randomized studies (MINORS)

	Studies		
**Criteria**	(32)	(38)	(39)
(1) A clearly stated aim: The question addressed should be precise and relevant in the light of available literature.	2	2	1
(2) Inclusion of consecutive patients: All patients potentially fir for inclusion (satisfying the criteria for inclusion) have been included in the study during the study period	2	1	2
(3) Prospective collection of data: Data were collected according to a protocol established before the beginning of the study.	1	2	2
(4) Endpoints appropriate to the aim of the study: Unambiguous explanation of the criteria used to evaluate the main outcome, which should be in accordance with the question addressed by the study. Also, the endpoints should be assessed on an intention-to-treat basis.	2	2	2
(5) Unbiased assessment of the study endpoint: Blind evaluation of objective endpoints and double-blind evaluation of subjective endpoints. Otherwise, the reasons for not blinding should be stated.	0	1	0
(6) Follow-up period appropriate to the aim of the study: The follow-up should be sufficiently long to allow the assessment of the main endpoint and possible adverse events.	1	1	1
(7) Loss to follow up less than 5%: All patient should be included in the follow up. Otherwise, the proportion lost to follow up should not exceed the proportion experiencing the major endpoint.	2	2	1
(8) Prospective calculation of study size: Information of the size of detectable difference of interest with a calculation of 95% confidence interval, according to the expected incidence of the outcome event, and information about the level for statistical significance and estimates of power when comparing outcomes.	1	2	2
9. All subjects for whom outcome measures were available received the treatment or control condition as allocated or, where this was not the case, data for at least one key outcome was analyzed by “intention to treat”	1	2	2
**Additional criteria in the case of comparative studies.**			
(9) An adequate control group: Having a gold standard diagnostic test or therapeutic intervention recognized as the optimal intervention according to the available published data.	2	-	-
(10) Contemporary groups: Control and studied group should be managed during the same time period (no historical controls)	2	-	-
(11) Baseline equivalence of groups: The groups should be similar regarding the criteria other than the studied endpoints. Absence of confounding factors that could bias the interpretation of results.	1	-	-
(12) Adequate statistical analyses: Whether the statistics were in accordance with the type of study with calculation of confidence intervals or relative risk.	2	-	-
**Total Score**:	19	15	15

## Analysis of the studies

The reviewed articles provided helpful insight into the effects of hippotherapy on the motor function of children with CP. Each study was equipped with perspicuous methodology and measurement tools to evaluate the outcomes that affect motor function. It is important to note that a common denominator in all articles was that the children had neither undergone surgery recently nor had been receiving any pharmaceutical treatment during the study process. The findings of every study are summarized below.

This study of Matusiak-Wieczorek et al. [32] involved a 12-week treatment program of hippotherapy combined with a traditional therapeutic approach. The findings demonstrated that the control of position and function of almost every assessed body part (head, trunk, feet, arms, hands) of the participants showed an improvement. Furthermore, when comparing changes in balance control and posture scores based on age, it was discovered that improvement occurred substantially more often in the intervention group, specifically in younger children aged 6–7 years (p = 0.001). Moreover, statistically significant improvement was more often observed among children who had frequent hippotherapy (twice a week). These improvements suggested the potential long-term benefits of hippotherapy. However, further research is required to validate these findings. It is important to note two key factors of uncertainty regarding the positive effect of hippotherapy, specifically the combined program of hippotherapy with a traditional therapeutic approach, as well as the small sample of participants.

In the article of Kwon et al. [33], the intervention program consisted of 30-minute sessions of hippotherapy, carried out twice a week for 8 consecutive weeks. The results of this study demonstrated significant improvements in motor function and balance of children who received hippotherapy intervention. More specifically, the Dimensions of GMFM-88 improved significantly after hippotherapy varied by GMFCS level: dimension E in level I, dimensions D and E in level II, dimensions C and D in level III, and dimensions B and C in level IV, and PBS measure, there was a large score increase in general. The findings of this study suggested that hippotherapy is a beneficial therapeutic approach.

Deutz et al. [34] study included two intervention periods of 16–20 weeks each, separated by a washout period of 16 weeks. The children were randomized to the early treatment group and the late treatment group, where the only difference was the timing of hippotherapy. Both groups received hippotherapy sessions once or twice weekly in addition to conventional physiotherapy. The study included multiple examination periods, treatment phases, and follow-up observations. The findings of this study exhibit that no significant changes were found in GMFM total scores, yet there was a notable increase in dimension E: walking, running, and jumping. On the other hand, no changes were observed regarding their quality of life. Altogether, the study indicated that hippotherapy and conventional therapeutic approaches positively affected gross motor function, especially the ability to walk, run, and jump.

Lucena-Antón et al. [35] investigated the effects of hippotherapy, combined with conventional therapy, on motor skills and hip spasticity in children with CP, and the intervention consisted of sessions lasting 45 min once a week for 12 consecutive weeks. The article’s findings indicate that the children who participated in combined intervention demonstrated a significant reduction of the spasticity in hip adductors due to the rhythmic, symmetrical movement of the horse that stimulated several systems simultaneously and caused mild dilation of the hip adductors. Overall, the outcome of this study suggested combining conventional therapy with hippotherapy at a specific frequency, as shown by other studies, to significantly improve hip adductor spasticity in children with CP and positively affect motor skills.

The scope of Chinniah et al. [36] was to investigate whether the Horse-Riding Simulator (HRS) is effective for children with spastic diplegia in sitting. The intervention group received 30-minute sessions of conventional physiotherapy and 15 min of HRS 3 times a week for 12 consecutive weeks. The main findings of this study show that, according to the scale, both groups gradually improved their sitting motor function in different stages of the treatment. Furthermore, the ones who received the combined therapy improved even better during the research period. Riding the HRS appeared to improve postural control in sitting and motor function of children with spastic diplegia due to the continuous and rhythmic muscular contractions on the rider produced by the simulator. In summary, further studies were considered mandatory to explore the effects of HRS on other motor functions of children with CP and to corroborate these findings.

Hemachithra et al. [37] aimed to investigate the immediate effects of HRS on adductor spasticity in children with spastic diplegia. The children were divided into two groups: the intervention group received one session of HRS for 30 min, while the other group was placed in a corner seat in a comfortable position, resting on pillows for 30 min. The outcome measures of this study were evaluated by MAS for adduction tone and a goniometer for hip abduction movement. This trial’s findings demonstrated that children subjected to HRS showed a reduction in muscle tone and an improvement in hip abduction range of motion. It should be emphasized that this study specifically focused on the immediate effects of HRS on abductor spasticity in children with CP. In conclusion, HRS seems to be a promising alternative therapeutic approach that can be integrated into physical therapy interventions.

Furthermore, the aim of the study of Champagne et al. [38] was to investigate the potential benefits of hippotherapy regarding the motor function and physical performance of children with CP. During the 23-week research, four evaluation sessions were carried out –two in the beginning, i.e., before the intervention (T1-T1’), to establish the baseline measures, one right after the end of the 10-week intervention (T2) and one last time 10 weeks after the therapeutic program ended/was terminated (T3). The hippotherapy intervention lasted 10 weeks, and each session was about 30 min per week. The therapeutic sessions included different positions of increasing difficulty regarding posture and orientation. The study’s results on the baseline provided no differences in most of the scores of BOT2-SF and the dimensions of GMFM-88, proving the stability of data between evaluations T1 and T1’. However, some subtests of the BOT2-SF were different, indicating the instability of those measurements. Moreover, the findings reveal that in evaluation T2, there were significant improvements in some of the BOT2-SF’s subtests and the dimensions measured by GMFM-88. Lastly, the fourth evaluation provided no significant differences between the scores of T2 and T3 for any measurement. Overall, the results of the present study support that hippotherapy can benefit gross motor function in children at GMFCS levels I and II.

The study of Yeo Seung Mi et al. [39] was aimed to identify individual factors influencing the gross motor outcome of hippotherapy in children with cerebral palsy (CP). The children received 30 min of hippotherapy twice a week for 8 weeks. The results of the study revealed that GMFCS levels I and II compared with IV (odds ratio [OR] ¼ 6.83) and III compared with IV (OR ¼ 4.45) were significantly associated with a good response to hippotherapy. In addition, higher baseline GMFM E (OR ¼ 1.05) and lower baseline GMFM B (OR ¼ 0.93) were also significantly associated with a good response to hippotherapy. Sex, age, CP type, and distribution were not factors influencing the gross motor outcome of hippotherapy. In conclusion, regarding the findings, the children with CP, GMFCS level I–III, with relatively poor postural control in sitting, might have a greater chance to improve their GMFM-66 scores through hippotherapy. This supports the hypothesis that hippotherapy is a context-focused therapy to improve postural control in sitting.

The aim of the study of Ali et al. [40] was to compare the efficacy of hippotherapy and whole-body vibration in ameliorating abdominal muscle thickness and sitting function in children with diplegia. The study’s sample was randomly allocated into two groups. Group A received conventional physical therapy for 1 h in addition to whole-body vibration, whereas Group B received hippotherapy for 40 min for 12 weeks, three times per week. The study showed a significant improvement in abdominal muscle thickness and sitting function (*p* < 0.05) in both groups, and greater improvements were observed in group B. The study concluded that whole-body vibration and hippotherapy training may be recommended to facilitate sitting function and ameliorate abdominal thickness in children with diplegia. Hippotherapy is more effective than whole-body vibration in improving sitting function and abdominal muscle thickness.

The primary objective of Prieto et al. study [41] was to examine the potential disparities in the impact of hippotherapy, administered either once or twice a week, on the gross motor function and functional performance of children diagnosed with cerebral palsy. These children were randomly assigned to either a once-weekly group (*n* = 9) or a twice-weekly group (*n* = 11). Over a period of 16 weeks, the participants completed 30-minute hippotherapy sessions. The Gross Motor Function Measure and the Pediatric Evaluation of Disability Inventory were administered both at the beginning of the study (baseline) and after a period of 16 weeks. A notable time effect was found in both groups, with no significant interactions observed between the groups. The utilization of hippotherapy has been found to enhance the gross motor function and functional performance of children diagnosed with cerebral palsy, irrespective of the frequency of the sessions conducted on a weekly basis.

^*1*^ *Horse Riding Simulator (HRS) used. GMFM: Gross Motor Function Measure; MAS: Modified Ashworth Scale; PROM: hip abduction passive range of motion; CHQ-28: Child Health Questionnaire; PBS: Pediatric Balance Scale; SAS: The Sitting Assessment Scale; MAS: Modified Ashworth Scale; BOT2 -SF: Bruininks-Oseretsky test of motor proficiency.*

## Discussion

The aggregated features of the reviewed studies (Table 3) provide essential evidence, supporting the hypothesis that hippotherapy can be beneficial for improving motor function of children aged 2–14 years old with CP. The findings demonstrate improvements in various aspects of motor function – more specifically in gross motor function skills, balance, coordination, gait parameters, and muscle strength [32–40].The studies’ outcomes are short-term, with a time frame from the last treatment to the assessment for each study.

Hippotherapy seems to benefit the rider due to the rhythmical and repetitive movement of the horse’s back, which synchronously activates and coordinates various neural and muscle systems. Measured scores clearly indicate an improvement in balance [33], body posture control [36], and sitting motor function [36] of children. Specifically, among the functions affected, it is important to mention the benefits of postural control, range of motion, muscle strength, and balance of the trunk, head, and upper limbs. Children with CP often toil with maintaining postural balance, affecting their ability to sit independently and, generally, their physique [33, 36, 42, 43]. The horse’s three-dimensional, continuous, and repeated movements help the rider maintain an upright posture, eventually improving their sitting motor function [36]. Daily life activities that require functional independence can be influenced, which means that children with CP may benefit greatly from hippotherapy with respect to their well-being and overall quality of life [44]. Balance is a fundamental skill necessary for maintaining controlled positions, such as sitting or engaging in physical activities. It also facilitates motor skill development. Speech therapists mentioned that proper body posture and alignment are crucial during feeding. Postural abnormalities, such as hyperextension of the neck, are frequently observed in children with CP. This can lead to the airway’s opening, which negatively affects swallowing dynamics and, most importantly, the protection of the lower airways [8]. Mothers of children with CP could spend 3.5 h to 7.5 h per day feeding their children, thus improving through hippotherapy the aforementioned impairments could give parents valuable help [45].

At the same time, there was a significant reduction in hip adduction spasticity [35, 37]. Reduction of spasticity in hip adductor muscles is one of the essential factors in improving the standing and gait of children with CP [46]. In addition, the positive effects of hippotherapy on walking speed and gait parameters, such as length of stride, show increased motor control during walking [47]. This was consensually attributed to the coordinated activation of systems that affect neuromuscular structures useful for movement. It is also noteworthy that similarly beneficial results were measured in the two studies [36, 37] where an HRS was employed instead of an actual animal. For these reasons, surgical and medical techniques have frequently been employed, but these approaches are costly and require specialized expertise [46]. A horseback riding simulator could be a useful alternative for the improvement of the static and dynamic balance of children with CP [48].

Moreover, factors such as sex, age, CP type, and distribution were not factors influencing the gross motor outcome of hippotherapy [39]. Furthermore, a study found that the ones who received the combined therapy improved even better during the research period [36]. Four studies [32, 35, 36, 40] The studies used a 12-week intervention period, but the program extended from 8 weeks to 20 weeks. Regarding the frequency and duration of therapy sessions, most studies included two sessions per week of 30 min each or one hour for one session per week. The above number of therapy sessions and duration seems beneficial as the outcomes from the studies were positive for the children.

Despite the substantially positive findings of the studies, it is important to relay the main limitations discussed across the selected studies. The most frequent was the small size of the sample, the fact that hippotherapy as an intervention method was used in addition to other conventional therapies rather than a standalone treatment, and the absence of a strict protocol in every study. Furthermore, the lack of heterogeneity of CP symptoms across the children and the different levels of the disorder was evident across the studies. Finally, the variety of evaluation tools and outcome measures makes directly comparing findings among the studies difficult. Moreover, our study had limitations to be noticed. Specifically, this review study included only research articles and only in the English language. A new study should be taken into advance of these limitations and cover this subject in more languages. Other limitations of this study were the last eight [8] years’ cover of the literature. The comparison between HRS and real Horses should be mentioned as a limitation of the studies included. The studies included in this review aimed to examine the potential disparities in the impact of hippotherapy (HRS or real Horses) on gross motor function compared only to other therapies and not between HRS and real horses. Future studies should be designed to compare these two intervention approaches.

## Conclusions

According to the findings of the selected studies, hippotherapy shows promising signs as a therapeutic method used for the improvement of motor function of children with CP. The positive effects it bears can be observed beyond the immediate intervention period, with the reviewed studies suggesting long-term follow-up effects. However, it is crucial to note that in so far it is being proposed as a supplementary therapeutic method to the conventional interventions, such as traditional physiotherapy, to gain faster additional benefits. Nevertheless, its inclusion as a complementary treatment may provide a supplementary motivation for children, making the therapy sessions more entertaining for the participant, leading to increased engagement and more frequent participation in the sessions. It stands to reason however that this may not be the case in HRS-based sessions, hence this is a hypothesis that needs to be tested with further research.

## Future implications

In spite of the promising findings of the reviewed literature, all researchers agreed that further research is required, emphasizing on the small sample sizes, the limited variation in the populations’ characteristics and diversity in the usage of evaluation tools and lack of common ground research protocols and standardization of the findings reports. Furthermore, there’s the need to assess whether hippotherapy will bring about significant long-term changes in motor skills in children with CP and thus affect positively their quality of life overall, either as a standalone treatment or a part of other traditional therapies.

## Electronic supplementary material

Below is the link to the electronic supplementary material.

Supplementary Material 1

## Data Availability

No new data were created or analyzed in this study.

## Abbreviations

CP: Cerebral palsy
AHA: American Hippotherapy Association
GMFCS: Gross Motor Function Classification System
SAS: Sitting Assessment Scale
HRS: Horse Riding Simulator
GMFM: Gross Motor Function Measure
MAS: Modified Ashworth Scale
PROM: Hip abduction passive range of motion
CHQ-28: Child Health Questionnaire
PBS: Pediatric Balance Scale
MAS: Modified Ashworth Scale
BOT2 -SF: Bruininks-Oseretsky test of motor proficiency
