# Supplementary material for: Effects of hippotherapy on motor function of children with cerebral palsy: a systematic review study
Source: Ital J Pediatr. 2024 Sep 19;50:188. doi: 10.1186/s13052-024-01715-9 (PMC11414029; doi:10.1186/s13052-024-01715-9)
Supplement: Supplementary file 1 — Supplementary Material 1 [file 13052_2024_1715_MOESM1_ESM.docx]

Dear Editor-in-Chief,

This candidate manuscript entitled *"Effects of hippotherapy on motor function of children with cerebral palsy: a systematic review study"* is revised according to the Reviewers’ comments.

This manuscript, which is a systematic review, aimed to study the effects of hippotherapy, as an alternative therapy, on motor function of children with Cerebral Palsy (CP). We performed a systematic review of the latest literature on hippotherapy and CP and the criteria we used were specific keywords, publication date, age of the subjects/studied population, and article type. The outcome of our research resulted in ten (10) relevant studies.

The results demonstrate improvements in various aspects of motor function – more specifically in gross motor function skills, balance, coordination, gait parameters, and muscle strength. Two of ten studies used the Horse-Riding Simulator (HRS) with significant improvements in postural control in sitting, motor function, and hip abduction range of motion. In conclusion, hippotherapy can be beneficial for the improvement of motor function skills of children between 2-14 years of age with CP, affecting their well-being and their overall quality of life. It can have a positive effect either as a standalone treatment or as a part of other traditional therapies. Extended research is needed to assess whether hippotherapy may present significant long-term changes in motor skills in children with CP.

Moreover, with this letter, it is confirmed that the study is original and has neither been previously published nor it is currently under consideration for publication elsewhere.

This research is relevant to the scope of the Journal. We hope this manuscript will interest Clinicians and Professionals interested in CP. If this paper is accepted will probably become a reference point for future publications regarding children with CP.

Please address all correspondence concerning this manuscript to [pplotas@upatras.gr](mailto:pplotas@upatras.gr). Thank you in advance for your consideration and attention relevant to the presented work.

Sincerely,

Plotas Panagiotis

MED, PhD

Assistant Professor

Department of Speech-Language Therapy,

School of Health Sciences and Rehabilitation,

University of Patras, Patras, Greece

e-mail address: [pplotas@upatras.gr](mailto:pplotas@upatras.gr)
